# Genetically Determined Rheumatoid Arthritis May Not Affect Heart Failure: Insights from Mendelian Randomization Study

**DOI:** 10.5334/gh.1256

**Published:** 2023-08-11

**Authors:** Xueqi Lin, Miaomiao Zhou, Chunsheng Zhang, Jiming Li

**Affiliations:** 1 Department of Cardiology, Shanghai East Hospital, Tongji University School of Medicine, Shanghai, CN; 2 Department of Cardiology, Shanghai East Hospital of Clinical Medical College, Dalian Medical University, Dalian, CN; 3 Department of Cardiology, Shanghai East Hospital of Clinical Medical College, Nanjing Medical University, Nanjing, CN

**Keywords:** rheumatoid arthritis, heart failure, Mendelian randomization, causal association

## Abstract

**Background:**

Evidence from observational epidemiological studies indicated that rheumatoid arthritis (RA) increased the risk of heart failure (HF). However, there is a possibility that the correlation is not explained as a causative role for RA in the pathogenesis of HF. A two-sample Mendelian randomization (MR) framework was designed to explore the potential etiological role of RA in HF to identify the target to improve the burden of HF disease.

**Methods:**

To assess the causal association between RA and HF, we analyzed summary statistics from genome-wide association studies (GWASs) for individuals of European descent. Genetic instruments for RA were identified at a genome-wide significance threshold (p < 5 × 10^–8^). Corresponding data were obtained from a GWAS meta-analysis (95,524 cases and 1,270,968 controls) to identify genetic variants underlying HF. MR estimates were pooled using the inverse variance weighted method. Complementary analyses were conducted to assess the robustness of the results.

**Results:**

There was no evidence of a causal association between genetically predicted RA and HF [odds ratio (OR), 1.00; 95% confidence interval (CI), 0.99–1.02; *P* = 0.60]. Various sensitivity analyses suggested no pleiotropy detected (all *p* > 0.05).

**Conclusion:**

Our findings did not support the causal role of RA in the etiology of HF. As such, therapeutics targeted at the control of RA may have a lower likelihood of effectively controlling the occurrence of HF.

## Introduction

Heart failure (HF) is a common cardiovascular disease characterized by the impairment of ventricular filling or blood ejection [1]. The pathophysiology of HF is multifarious and complex, with a sophisticated interaction between genetic and environmental factors [2]. There is convincing evidence that inflammation plays a significant role in the pathogenesis of HF [3], and the comorbidity paradigm between HF and autoimmune diseases has been widely reported [4].

Rheumatoid arthritis (RA) is a systemic autoimmune polyarthritis with a core feature of abnormal activation of immunity [5,6]. While the joint disease per se is not lethal, about half to a third of premature deaths in patients with RA are due to an increased risk of cardiovascular events [7,8,9,10]. As the second leading cause of death in RA patients, HF is underappreciated [3]. An emerging body of literature indicates that RA patients are almost twice as likely to develop HF as individuals without RA [4,9], which could not be explained by an increased frequency or effect of cardiovascular risk factors and coronary artery disease [11,12]. Additionally, the time of HF onset is shorter in RA groups than in non-RA groups [9]. Therefore, RA should be considered a possible risk factor for HF. Traditionally, RA was presumed to be associated with an increased risk of HF due to inflammatory activity [3]. This hypothesis was further supported by some studies which suggested a link between comorbidities associated with systemic inflammation and the development of HF [13,14,15]. Nevertheless, recent randomized controlled trials seem to be contradictory and elusive in favor of the benefit of RA therapy for HF [3,9,16,17,18]. Thus, it is unclear whether RA and HF are associated.

With the limited suggestive evidence from observational studies, Mendelian randomization (MR) analysis offers an opportunity to clarify the potential causal association between RA and HF. MR is an efficient and reliable epidemiological research method [19,20] that uses genetic variants as a proxy for risk factors, mimicking the randomization assignment that underpins causal inference in randomized controlled trials [21,22]. In particular, MR capitalizes on the randomly allocated offspring allele inheritance that occurs naturally during the formation of zygote [23], which could minimize the inherent problems of confounding and eliminate reverse causality in observational studies [24,25].

Unraveling the causal links between RA and HF may provide new avenues for the management of HF. Here, we conducted a two-sample MR aimed to test the hypothesis that RA is causally related to the risk of HF from a genetic perspective.

## Materials and Methods

### Study design

MR techniques use genetic variants as instrumental variables (IVs) to mimic the biological effects that can enhance causal inference [26,27]. Three key assumptions must hold for the MR study to be valid [20,28]: (i) each genetic variant is highly associated with risk factor (the relevance assumption); (ii) no significant association is observed between the genetic variant and the confounders of the risk factor-outcome relationship (the independence assumption); (iii) each genetic variant does not have any association with the outcome conditional on the risk factor and the confounders of the risk factor-outcome relationship (the exclusion restriction). If all these assumptions are met, then the only causal pathway from the genetic variant to the outcome is the risk factor, and there are no other causal pathways, either directly to the outcome or via confounders (supplementary material *Figure S1*). We estimated the genetically predicted effects of RA on HF using a two-sample MR framework [29].

### Data source

The datasets used in our MR analyses were derived from publicly available genome-wide association studies (GWASs). Only participants of European ancestry were included in order to reduce population stratification bias and improve the stability of the analyses. Detailed information on all summary statistics used in the MR study is provided in supplementary materials *Table S1*. There was no sample overlap between GWAS for RA and HF.

We were exposed to genetically determined RA, as IVs and HF were the primary outcome. RA-associated variants were extracted from the Okada et al. [30]. All cases were defined using electronic health records (ICD-9 714.0, ICD-10: M05, or M06). Several aspects of the study design, such as the collection of samples, quality control procedures, and imputation methods, have been described in the original article. Levin et al. provided a summary of the genetic variants’ association with HF following the identification of the genetic variants [31]. GWAS summary statistics for HF were obtained from non-overlapping analyses of six separate cohorts/consortia. HF was defined using cohort-specific definitions (pheCodes80 or *ICD-9/10* codes documented within the electronic health record for all studies except HERMES, which additionally included expert adjudication among some cohorts).

In this study, only summaries of published studies were utilized, and individual data were not involved. All studies contributing data to these analyses were approved by relevant ethics committees, and all participants provided written informed consent.

### IVs selection and quality control

We retrieved summary estimations of genetic variants to be associated with RA in the Okada et al. study and identified reached genome-wide significance (*p* < 5 × 10^–8^). For the purpose of ensuring genetic independence among variants, the threshold of linkage disequilibrium (LD) was set to r^2^ < 0.001 and located at 10,000 kb for further pruning [24]. We screened the secondary phenotypes of each selected instrument in PhenoScanner V2, a database of human genotype-phenotype associations [32,33]. Harmonization was conducted to eliminate strand mismatches and ensure that the SNP effect sizes on both RA and HF correspond to the same allele.

### Statistical analyses

A two-sample MR approach was performed after harmonizing summary data based on a previously described method [34]. We used a set of complementary methods for the robustness of the results. The inverse variance weighted (IVW) method of the random effects model assumes that either all IVs are valid, or any horizontal pleiotropy is balanced [35]. The weighted median method can generate an unbiased estimate if at least 50% of the weight comes from valid IVs [36], and the MR-Egger regression method is primarily used to account for potential horizontal pleiotropy by its intercept [37]. In addition, the Mendelian Randomization Pleiotropy RESidual Sum and Outlier (MR-PRESSO) method was employed to identify potential outliers and correct, if necessary, for possible horizontal pleiotropic outliers in the analysis [38]. We used a pre-defined approach for selecting the best statistical estimate among these four methods (Supplement material *Figure S2*) and considered the association as casual when at least three methods provided consistent results. A leave-one-out analysis was conducted by removing every single variant from the analysis without exception. The fluctuation of the estimates in response to the exclusion of each variant reflects the potential of an outlier variant in the causal estimate. Additionally, Cochran’s *Q* statistics were used to examine the global heterogeneity across IV-specific MR estimates [39]. These sensitivity analyses were useful for determining whether a causal conclusion from such an analysis was plausible or not.

All the statistical analyses were carried out in the R program (version 4.2.1), using the meta package (version 6.0–0), the TwoSampleMR package (version 0.5.6), the MRPRESSO package (version 1.0) and the MendelianRandomization package (version 0.6.0). Statistical significance association is defined as a *p*-value < 0.05.

## Results

We extracted 46 independent SNPs that reached genome-wide significance from RA (Supplementary material *Table S2*). Most SNPs were available in the GWAS of HF except for two (rs3799963, rs1042169). In the PhenoScanner and GWAS catalog, we identified four selected SNPs that were associated with confounders (Supplementary material Table S2). We removed one SNP for being palindromic. Characteristics of the genetic association of IVs with RA and HF outcomes are shown in Supplementary *Table S3*. According to the IVW analysis, genetically predicted RA was not associated with HF (Table 1; Supplementary material Table S3; Supplementary material Figure S4). The causal association between RA and HF was confirmed using the weighted median, MR-PRESSO, MR-Egger regression, and leave-one-out methods (Table 1; Supplementary material *Table S4*, Supplementary material *Figure S3*) in the complementary analyses. Importantly, the MR-MRESSO method detected two outliers, but the results were similar after excluding the outliers (Table 1, Supplementary material *Table S5*).

**Table 1:** Two-sample Mendelian randomization estimates between rheumatoid arthritis and heart failure.


OUTCOME	SNP SELECTION	SNPS,N	METHOD	OR (95% CI)	P-VALUE

HF	All	39	IVW	1.00(0.99–1.02)	0.68

			MR Egger	1.01(0.99–1.03)	0.37

			Weighted median	1.01(0.99–1.02)	0.38

			Weighted mode	1.00(0.99–1.02)	0.30

	Remove	37	IVW	1.00(0.99–1.01)	0.62

			MR Egger	1.01(0.99–1.03)	0.31

			Weighted median	1.01(0.99–1.02)	0.37

			Weighted mode	1.01(0.99–1.02)	0.36


Abbreviation: HF, heart failure; SNPs, single nucleotide polymorphisms; IVW, Inverse Variance Weighted; MR-Egger, Mendelian Randomization-Egger; OR, odd ratios; CI, confidence interval.

## Discussion

HF is a complex clinical syndrome with a multitude of potential risk factors and causes. The observation that RA may be associated with HF leads us to seek further evidence to confirm this hypothesis. In contrast to previous observational studies, our MR analysis of the European population did not provide evidence that genetically predicted RA contributes to an increased risk of HF.

RA and HF share similar inflammatory pathologies [3,40]. According to Mantel et al., in 10,000 Swedish patients with RA, the risk of HF increased rapidly after the onset of the disease and was associated with a high level of activity [9]. When stratified by HF subtype, C-reactive protein levels accounted for a greater proportion of HF with preserved ejection fraction than HF with reduced ejection fraction, indicating that inflammation may be a great contributor to the former in RA patients [41]. In patients with RA who do not have overt cardiovascular disease, local inflammation in the myocardium may be evident on cardiac magnetic resonance imaging [42]. Some studies have suggested that RA is causally related to hypertension, age-related heart attack, and coronary heart disease [43,44]. In our study, however, there was no direct causal relationship between RA and HF. HF can be caused by a variety of factors, including activation of the neurohumoral system, changes in blood pressure, or changes in ventricular hemodynamics [45]. In addition to inflammation, oxidative stress (as a marker of an imbalance between reactive oxygen species and antioxidants) is also typically increased and contributes to the development of HF and RA, representing another potentially shared pathway between the diseases [46]. Unsurprisingly, RA patients with increased levels of disease activity and systemic inflammation are at an increased risk of developing HF with preserved ejection fraction [47]. While the results of our study indicated no causal relationship between RA and HF incidence, RA may influence the progression of HF by increasing various inflammatory factors, which is beyond the scope of the current study. It is also important to note that some literature suggests that specific ‘treat-to-target’ RA therapies may not always be beneficial for the prevention of HF, and the mechanisms warrant further research [10,40].

We have contributed to the rethinking of intervention targets for reversing RA and preventing HF as a result of our study. Compared to clinical trials, MR is a more cost-effective, quicker, and more ethical method of evaluating the long-term effects of interventions on RA. It is particularly important since efforts are still being made to develop interventions that can reverse complications related to RA.

To the best of our knowledge, this is the first MR study to explore the relationship between RA and HF. However, limitations should be considered when interpreting the results of this study, as RA and HF could be connected via complex biological pathways. The limited IVs may take current MR analyses underpowered. Since the databases used in the MR analysis were conducted on participants of European ancestry, the results may be biased and may not apply to other races. In the absence of individual data, it was not possible to conduct stratified analyses of disease activity or HF subtypes and severity. Moreover, we excluded SNPs associated with systemic lupus erythematosus or hyperthyroidism, but there may still be other potential confounders that could violate the independence assumption, and it was not feasible to exclude them all.

As a result, the MR study does not support a causal relationship between RA and HF in the current MR analyses. It was only from a genetic perspective that we investigated the causal relationship between certain forms of RA and HF. Therefore, our results should be treated with caution since the causal effect of genetic variants exposure on the outcome can be modified by compensatory processes during development. Further research with larger sample sizes is necessary to provide a more accurate estimate.

## Conclusion

Our MR analysis did not identify convincing evidence to support the causal relationship between RA with HF. A larger sample size is needed to confirm our MR result further.

## Additional File

The additional file for this article can be found as follows:

10.5334/gh.1256.s1 Supplementary Materials. Supplementary Figures S1 to S4 and Tables S1 to S5.
