# Supplementary material for: Genetically Determined Rheumatoid Arthritis May Not Affect Heart Failure: Insights from Mendelian Randomization Study
Source: Glob Heart. 2023 Aug 11;18(1):43. doi: 10.5334/gh.1256 (PMC10418047; doi:10.5334/gh.1256)

Supplementary Materials

Genetically Determined Rheumatoid Arthritis may not Affect Heart Failure: Insights from Mendelian Randomization Study

Xueqi Lin, Miaomiao Zhou, Chunsheng Zhang, Jiming Li

Figure S1: Directed acyclic graph of the Mendelian randomization assumptions in this study
Table S1: Contributing studies and information on the datasets used for analysis
Figure S3: Predefined decision tree of the selection of methods to evaluate associations using two-sample Mendelian randomization
Table S2: Related traits of rheumatoid arthritis-associated SNPs
Table S3: Harmonized dataset of two-sample Mendelian randomization for the effect of rheumatoid arthritis on heart failure
Table S4: Heterogeneity and horizontal pleiotropy analyses between rheumatoid arthritis and heart failure
Table S5 SNP exluded from the outlier corrected MR-MRPRESSO analyses between rheumatoid arthritis and heart failure
Figure S3: Leave-one-out plot to assess if a single variant is driving the association between rheumatoid arthritis and heart failure
Figure: S4: Forest plots of variants specific inverse variance estimates for the casual association between rheumatoid arthritis and heart failure

Figure S1: Directed acyclic graph of the Mendelian randomization framework in this study

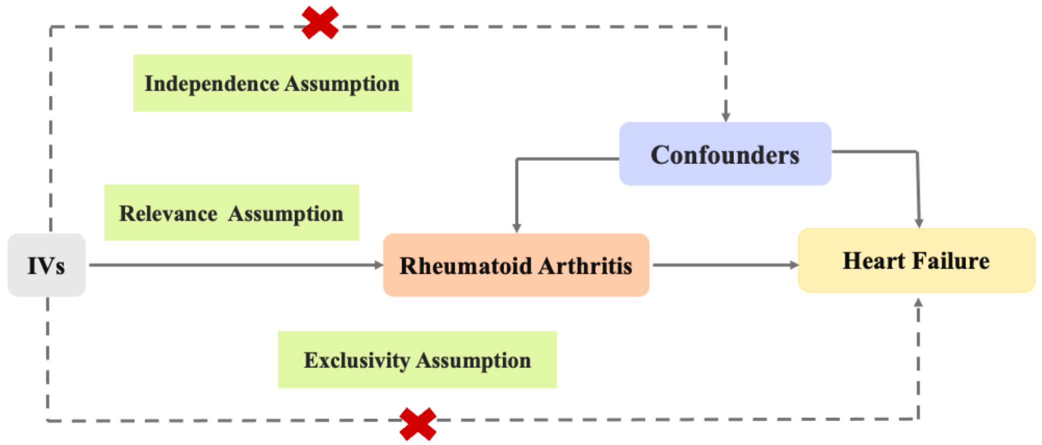

If the genetic variant satisfied three key assumptions of an instrumental variable (IV) for the risk factor, then candidate IV is particularly appropriate for testing whether the risk is the cause of the outcome since it is fixed at conception and cannot be affected by environmental factors that would otherwise result in confounding or reverse causation. IVs used in our MR analyses are satisfied with three key assumptions which imply the only causal pathway from the genetic variant to the outcome is via the risk factor, and there is no other causal pathway either directly to the outcome or via a confounder

Table S1 Contributing studies and more information on the datasets used for analyses

Use in MR	Phenotype	Data source	nCases	Sample size	Contributing studies	Sample overlap
**Exposure**	RA	Okada et al.	14,361	58,284	BRASS, CANADA, EIRA, NARAC1, NARAC2, WTCCC, Rheumatoid Arthritis Consortium International for Immunochip (RACI)-UK, RACI-US, RACI-SE-E, RACI-SE-U, RACI-NL, RACI-ES, RACI-i2b2, ReAct, Dutch (including AMC, BeSt, LUMC, and DREAM), anti-TNF response to therapy collection (ACR-REF: BRAGGSS, BRAGGSS2, ERA, KI, and TEAR), CORRONA, Vanderbilt,	0
**Outcome**	HF	Levin et al	95,524	1,366,492	HERMES,DISCOVEHR,EMERGE,FINNGEN,BIOME,PMBB	0

Abbreviation: MR, Mendelian randomization; RA, rheumatoid arthritis; HF, heart failure

Figure S2: Predefined decision tree of the selection of methods to evaluate associations using two-sample Mendelian randomization

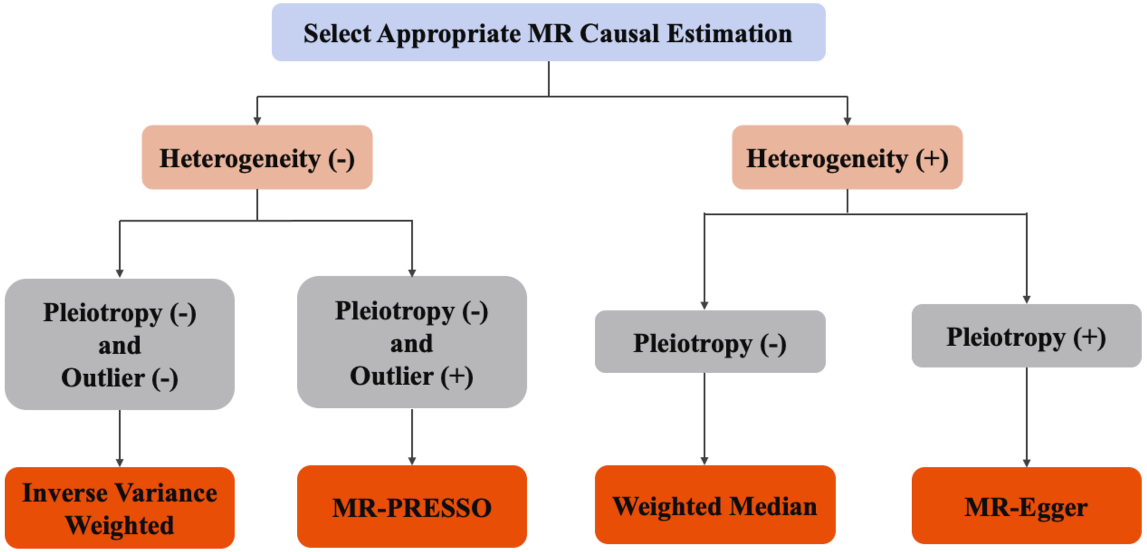

Abbreviation: MR, Mendelian randomization; MR-PRESSO, Mendelian Randomization Pleiotropy RESidual Sum and Outlier

Table S2 Related traits of Rheumatic arthritis-associated SNPs

SNP	Chr	Pos	EA	NEA	Trait	Excluded from complementary analysis
**rs61828284**	1	173299743	T	C	Rheumatoid arthritis	No
**rs60733400**	1	2516781	A	G	Rheumatoid arthritis	No
**rs2240336**	1	17674402	T	C	Rheumatoid arthritis	No
**rs28411352**	1	38278579	T	C	Rheumatoid arthritis	No
**rs6679677**	1	114303808	A	C	Rheumatoid arthritis ; Basophil percentage of white cells; Crohns disease; Hypothyroidism; Juvenile idiopathic arthritis including oligoarticular and rheumatoid factor negative polyarticular JIA; Type 1 diabetes; Antineutrophil cytoplasmic antibody associated vasculitis; Pediatric autoimmune diseases; Systemic lupus erythematosus; Self-reported hyperthyroidism or thyrotoxicosis; Self-reported hypothyroidism or myxoedema	Yes (Systemic lupus erythematosus and hyperthyroidism or thyrotoxicosis)
**rs13426947**	2	191933254	A	G	Rheumatoid arthritis; Self-reported hypothyroidism or myxoedema	No
**rs3087243**	2	204738919	A	G	Alopecia areata; Graves disease; Rheumatoid arthritis; Selective immunoglobulin A deficiency IgAD; Type 1 diabetes; Self-reported hyperthyroidism or thyrotoxicosis; Self-reported hypothyroidism or myxoedema	Yes (hyperthyroidism or thyrotoxicosis)
**rs9653442**	2	100825367	T	C	Rheumatoid arthritis; Intelligence multi trait analysis; Years of educational attainment	No
**rs34695944**	2	61124850	C	T	Rheumatoid arthritis	No

**rs2661798**	2	65635688	T	A	Rheumatoid arthritis; White blood cell count	No
**rs9310852**	3	27784997	G	A	Rheumatoid arthritis; Lymphocyte count	No
**rs4452313**	3	17047032	T	A	Rheumatoid arthritis	No
**rs73081554**	3	58302935	T	C	Rheumatoid arthritis	No
**rs34046593**	4	26111593	A	G	Rheumatoid arthritis; Self-reported hypothyroidism or myxoedema	No
**rs7731626**	5	55444683	A	G	Pediatric autoimmune diseases; Rheumatoid arthritis	No
**rs2561477**	5	102608924	A	G	Rheumatoid arthritis; Self-reported hypothyroidism or myxoedema	No
**rs62395855**	6	31884909	T	C	Lymphocyte count; Body fat percentage; Rheumatoid arthritis; Trunk fat percentage	No
**rs56339890**	6	29782742	T	C	Eosinophil percentage of white cells; Rheumatoid arthritis; Hemoglobin concentration; Reticulocyte count	No
**rs2844456**	6	31864674	C	T	Granulocyte count; Asthma; Self-reported hypothyroidism or myxoedema; Self-reported malabsorption or coeliac disease; Hemoglobin concentration; Rheumatoid arthritis; Intestinal malabsorption; Hemoglobin concentration; Lymphocyte count; Neutrophil count; Myeloid white cell count; Peak expiratory flow	No
**rs6936656**	6	32393281	T	C	Height; Psoriasis; Self-reported hyperthyroidism or thyrotoxicosis; Self-reported malabsorption or coeliac disease; Self-reported psoriasis; Self-reported psoriatic arthropathy; Rheumatoid arthritis	Yes (hyperthyroidism or thyrotoxicosis)
**rs17264332**	6	138005515	G	A	Celiac disease; Inflammatory bowel disease; Rheumatoid arthritis; Self-reported malabsorption or coeliac disease;	No

					Neutrophil percentage of white cells; Lymphocyte percentage of white cells	
**rs62401699**	6	31022928	T	C	Granulocyte count; IgA deficiency; Height; Rheumatoid arthritis	No
**rs71565347**	6	32722961	A	G	Rheumatoid arthritis; Self-reported psoriasis	No
**rs1571878**	6	167540842	T	C	Rheumatoid arthritis; Self-reported hypothyroidism or myxoedema	No
**rs9275183**	6	32654502	G	A	Body mass index males; Sum eosinophil basophil counts; IgA deficiency; Inflammatory bowel disease; Ulcerative colitis; Primary sclerosing cholangitis; Asthma; Nasal polyp; Long-standing illness, disability or infirmity; Intestinal malabsorption; Self-reported ulcerative colitis; Self-reported malabsorption or coeliac disease; Self-reported polymyalgia rheumatica	No
**rs1042169**	6	33048686	A	G	NA	No
**rs212389**	6	159489791	A	G	Granulocyte count; Rheumatoid arthritis; Myeloid white cell count; Neutrophil count	No
**rs12539741**	7	128596805	T	C	Rheumatoid arthritis; Systemic lupus erythematosus	Yes (Systemic lupus erythematosus)
**rs11574914**	9	34710338	A	G	NA	No
**rs3799963**	6	44231479	C	G	NA	No
**rs10985070**	9	123636121	A	C	Hayfever, allergic rhinitis or eczema; Rheumatoid arthritis; Lymphocyte count	No
**rs12764378**	10	63800004	A	G	Rheumatoid arthritis; Self-reported hypothyroidism or myxoedema; Trunk fat-free mass; Trunk predicted mass	No

**rs706778**	10	6098949	T	C	Alopecia areata; Rheumatoid arthritis; Primary sclerosing cholangitis; Pediatric autoimmune diseases; Self-reported hypothyroidism or myxoedema	No
**rs537544**	10	8108382	T	C	Rheumatoid arthritis; Asthma	No
**rs10790268**	11	118729391	G	A	Allergic disease; Rheumatoid arthritis	No
**rs9603608**	13	40318819	C	A	Eosinophil count; Rheumatoid arthritis; Neutrophil percentage of granulocytes	No
**rs8032939**	15	38834033	C	T	Rheumatoid arthritis; Self-reported hypothyroidism or myxoedema; Eosinophil percentage of white cells	No
**rs8026898**	15	69991417	A	G	Rheumatoid arthritis; Height	No
**rs13330176**	16	86019087	A	T	Rheumatoid arthritis; Monocyte count	No
**rs12232497**	17	38040119	C	T	Allergic disease; High density lipoprotein; Asthma; Primary biliary cirrhosis; Crohns disease; Ulcerative colitis; Inflammatory bowel disease; Rheumatoid arthritis	No
**rs592390**	18	12822314	C	T	Rheumatoid arthritis	No
**rs34536443**	19	10463118	C	G	Juvenile idiopathic arthritis including oligoarticular and rheumatoid factor negative polyarticular JIA; Psoriasis; Self-reported hypothyroidism or myxoedema; Rheumatoid arthritis	No
**rs4239702**	20	44749251	C	T	Rheumatoid arthritis	No
**rs8133843**	21	36738242	A	G	Rheumatoid arthritis, Eosinophil percentage of white cells	No
**rs225433**	21	43809418	G	C	Rheumatoid arthritis	No
**rs2069235**	22	39747780	A	G	Primary biliary cirrhosis; Weight; Rheumatoid arthritis	No

Abbreviation: SNP, single nucleotide polymorphis; Chr, chromosome; Pos, Position; EA, Effect allele; NEA, non Effect allele
Evidence of association ($p<5\times10^{-8}$) of the SNPs used as genetic variants for Mendelian randomization analyses of rheumatoid arthritis with confounders or heart failure s in the PhenoScanner and the GWAS catalog.

Table S3 Harmonized dataset of two-sample Mendelian randomization for the effect of rheumatoid arthritis on heart failure

SNP	Chr	Pos (GRCh38)	EA	NEA	EAF	RA			HF			÷R^2 (%)	φ*F*-statistic
						β	SE	*p*-value	*β*	SE	*p*-value		
rs10790268	11	118729391	G	A	0.81	0.16	0.021	3.30E-15	-0.010	0.0065	0.12	0.80	475.56
rs10985070	9	123636121	A	C	0.57	-0.083	0.015	1.70E-08	-0.0091	0.005	0.071	0.30	199.80
rs11574914	9	34710338	A	G	0.32	0.12	0.017	1.50E-13	-0.0051	0.0058	0.38	0.60	378.20
rs12232497	17	38040119	C	T	0.47	0.094	0.016	3.60E-09	-0.017	0.0051	0.00099	0.40	257.70
rs12764378	10	63800004	A	G	0.25	0.13	0.018	1.90E-13	-0.0044	0.0061	0.48	0.60	372.54
rs13330176	16	86019087	A	T	0.24	0.11	0.020	9.00E-09	-0.0022	0.0060	0.72	0.50	274.04
rs13426947	2	191933254	A	G	0.19	0.13	0.019	2.40E-12	0.0028	0.0061	0.64	0.50	310.75
rs1571878	6	167540842	T	C	0.58	-0.12	0.015	4.90E-15	-0.0066	0.005	0.19	0.70	388.43
rs17264332	6	138005515	G	A	0.17	0.16	0.018	7.10E-19	-0.0019	0.0067	0.77	0.70	435.61
rs2069235	22	39747780	A	G	0.30	0.10	0.017	3.00E-10	0.0068	0.0056	0.22	0.50	267.41
rs212389	6	159489791	A	G	0.63	0.095	0.016	1.10E-09	0.0010	0.0055	0.86	0.40	247.87
rs2240336	1	17674402	T	C	0.42	-0.11	0.017	1.40E-09	0.0066	0.0051	0.19	0.50	315.73
rs225433	21	43809418	G	C	0.19	-0.13	0.023	1.80E-08	0.0012	0.0060	0.84	0.50	293.44

rs2561477	5	102608924	A	G	0.30	-0.11	0.017	5.20E-10	-0.018	0.0055	0.0012	0.50	274.05
rs2661798	2	65635688	T	A	0.45	0.094	0.015	1.10E-09	0.015	0.0052	0.0050	0.40	257.56
rs28411352	1	38278579	T	C	0.25	0.10	0.018	5.20E-09	-0.0043	0.0057	0.45	0.40	239.33
rs2844456	6	31864674	C	T	0.04	0.89	0.029	1.00E-200	0.012	0.014	0.39	6.10	3789.59
rs34046593	4	26111593	A	G	0.32	0.14	0.017	9.20E-17	-0.0074	0.0059	0.21	0.90	500.61
rs34536443	19	10463118	C	G	0.028	-0.38	0.047	4.60E-16	-0.0074	0.015	0.62	0.80	457.90
rs34695944	2	61124850	C	T	0.36	0.12	0.015	4.40E-14	-0.0095	0.0056	0.090	0.60	367.37
rs4239702	20	44749251	C	T	0.71	0.14	0.018	4.20E-14	-0.0095	0.0056	0.089	0.80	466.59
rs4452313	3	17047032	T	A	0.31	0.11	0.017	2.70E-10	0.013	0.0053	0.017	0.50	277.20
rs537544	10	8108382	T	C	0.62	-0.12	0.018	8.00E-11	0.0057	0.0052	0.27	0.60	375.23
rs56339890	6	29782742	T	C	0.020	-0.40	0.065	7.10E-10	-0.023	0.023	0.32	0.60	368.74
rs592390	18	12822314	C	T	0.48	-0.095	0.016	3.80E-09	-0.0047	0.0052	0.37	0.50	265.37
rs60733400	1	2516781	A	G	0.31	-0.12	0.017	1.30E-09	-0.0012	0.0056	0.82	0.50	280.11
rs61828284	1	173299743	T	C	0.080	-0.20	0.034	8.70E-09	0.016	0.011	0.14	0.60	337.89
rs62395855	6	31884909	T	C	0.020	-0.62	0.065	4.40E-21	-0.036	0.018	0.040	1.50	880.56

rs62401699	6	31022928	T	C	0.040	-0.24	0.041	1.30E-08	0.0088	0.013	0.48	0.40	249.78
rs706778	10	6098949	T	C	0.41	0.10	0.015	7.10E-12	-0.0025	0.005	0.62	0.50	309.51
rs71565347	6	32722961	A	G	0.020	-0.46	0.071	9.80E-11	0.012	0.019	0.52	0.80	491.84
rs73081554	3	58302935	T	C	0.052	0.17	0.030	4.70E-08	-0.0066	0.011	0.55	0.30	156.98
rs7731626	5	55444683	A	G	0.38	-0.20	0.020	7.90E-23	0.0020	0.0057	0.73	1.90	1099.34
rs8026898	15	69991417	A	G	0.29	0.15	0.018	2.40E-17	0.0022	0.0058	0.71	0.90	528.32
rs8032939	15	38834033	C	T	0.024	0.12	0.017	2.40E-12	0.0078	0.0056	0.16	0.10	37.25
rs8133843	21	36738242	A	G	0.63	0.095	0.016	6.00E-09	-5.00E-04	0.0051	0.92	0.40	249.18
rs9275183	6	32654502	G	A	0.16	0.78	0.023	1.00E-200	0.0054	0.0068	0.43	16.2	11274.04
rs9310852	3	27784997	G	A	0.46	0.083	0.015	3.20E-08	-0.0044	0.0052	0.40	0.30	202.14
rs9603608	13	40318819	C	A	0.34	-0.104	0.016	7.60E-11	-0.0033	0.0053	0.53	0.50	287.86
rs9653442	2	100825367	T	C	0.55	-0.11	0.015	3.60E-12	-8.00E-04	0.0050	0.87	0.60	322.44

Abbreviation: SNP, single nucleotide polymorphism; Chr, chromosome; Pos, Position; EA, Effect allele; NEA, non Effect allele; EAF, effect allele frequency; SE, standard error; RA, rheumatoid arthritis; HF, heart failure; ⁺R^2, explained variation by SNPs; ᵠ*F*-statistic, the strength of the IV-exposure correlation

Table S4 Heterogeneity and horizontal pleiotropy analyses between rheumatoid arthritis and heart failure

**SNP**	**Heterogeneity**			**Horizontal pleiotropy**			**MR-PRESSO**	**Power estimation (power= 80%)**
	**IVW Q**	**IVW Q df**	***p*-value**	**Egger intercept**	**SE**	***p*-value**		
ALL	62.91	38	0.0067	-0.0016	0.0020	0.41	0.99	1.04 (0.96)
Removed	41.70	36	0.24	-0.0016	0.0017	0.36	NA	

Abbreviation: IVW Q, Inverse variance weighted Q Cochran's Q test estimate; IVW Q df, Inverse variance weighted Q Cochran's Q test degrees of freedom; SE, standard error; MR-PRESSO, Mendelian Randomization Pleiotropy RESidual Sum and Outlier

Table S5 SNP exluded from the outlier corrected MR-MRPRESSO analyses between rheumatoid arthritis and heart failure

SNP	RSSobs	*p*-value	SNP	RSSobs	*p*-value
**rs10790268**	1.16E-04	1	**rs4239702**	1.02E-04	1
**rs10985070**	7.98E-05	1	**rs4452313**	1.57E-04	0.6786
**rs11574914**	3.05E-05	1	**rs537544**	3.75E-05	1
**rs12232497**	2.97E-04	0.0039	**rs56339890**	4.94E-04	1
**rs12764378**	2.35E-05	1	**rs592390**	2.00E-05	1
**rs13330176**	6.52E-06	1	**rs60733400**	8.19E-07	1
**rs13426947**	6.02E-06	1	**rs61828284**	2.80E-04	1
**rs1571878**	4.05E-05	1	**rs62395855**	1.26E-03	1
**rs17264332**	5.81E-06	1	**rs62401699**	9.18E-05	1
**rs2069235**	4.31E-05	1	**rs706778**	8.05E-06	1
**rs212389**	5.34E-07	1	**rs71565347**	1.92E-04	1
**rs2240336**	4.89E-05	1	**rs73081554**	5.07E-05	1
**rs225433**	2.53E-06	1	**rs7731626**	7.11E-06	1
**rs2561477**	3.09E-04	0.0429	**rs8026898**	3.26E-06	1
**rs10790268**	1.16E-04	1	**rs8032939**	5.71E-05	1
**rs28411352**	2.16E-05	1	**rs8133843**	6.14E-07	1
**rs2844456**	1.16E-04	1	**rs9275183**	2.58E-05	1
**rs34046593**	6.29E-05	1	**rs9310852**	2.19E-05	1
**rs34536443**	4.13E-05	1	**rs9603608**	9.20E-06	1
**rs34695944**	9.92E-05	1	**rs9653442**	2.52E-07	1

Abbreviation: SNP, single nucleotide polymorphism

Figure S3: Leave-one-out plot to assess if a single variant is driving the association between rheumatoid arthritis and heart failure

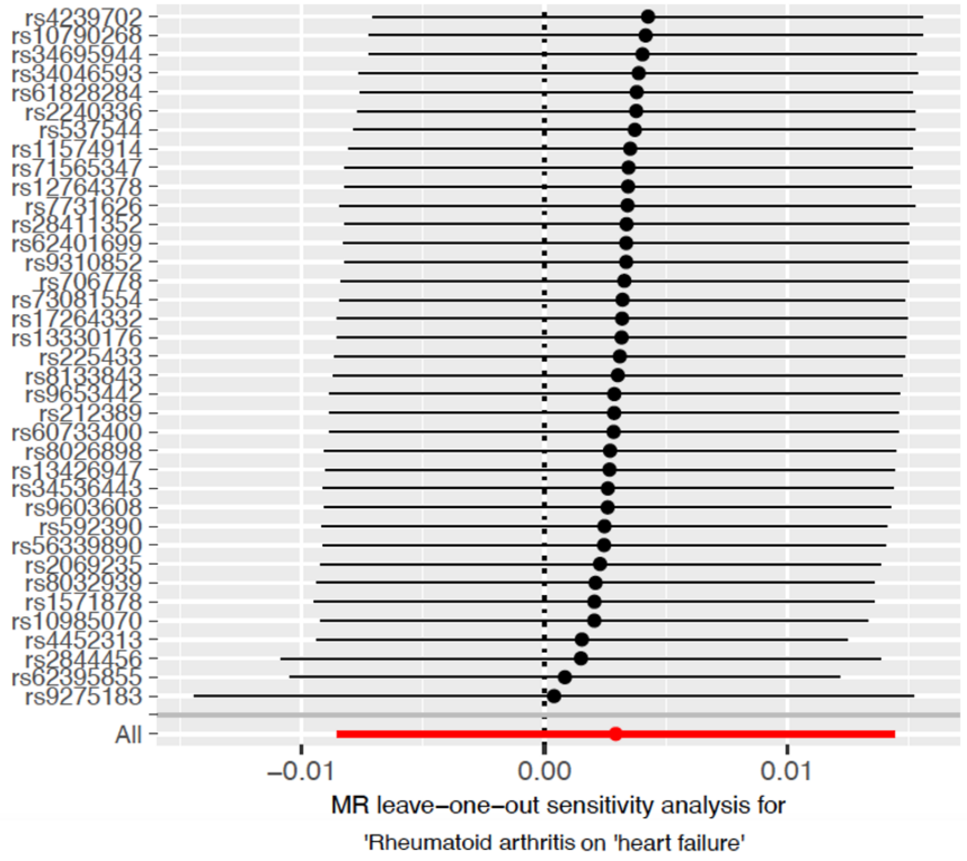

Figure S4: Forest plots of variants specific inverse variance estimates for the casual association between rheumatoid arthritis and heart failure

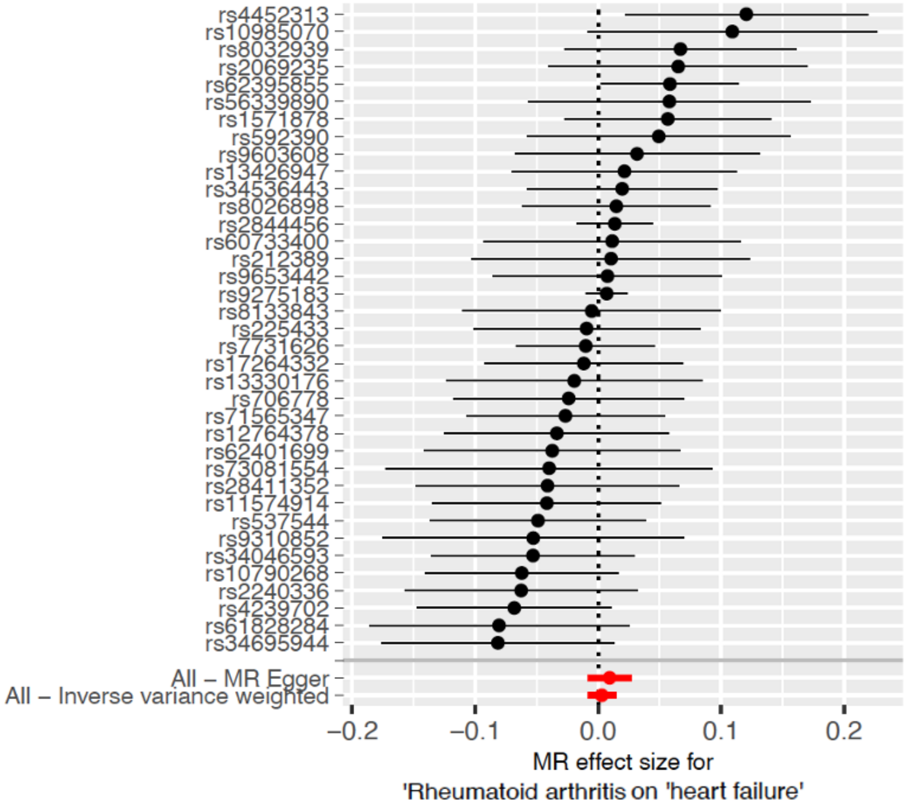

Supplement: Supplementary Materials. — Supplementary Figures S1 to S4 and Tables S1 to S5. [file gh-18-1-1256-s1.pdf]
